# Involvement of Secretin in the Control of Cell Survival and Synaptic Plasticity in the Central Nervous System

**DOI:** 10.3389/fnins.2020.00387

**Published:** 2020-05-06

**Authors:** Lei Wang, Li Zhang

**Affiliations:** ^1^School of Life Sciences, Guangzhou University, Guangzhou, China; ^2^GHM Institute of CNS Regeneration, Jinan University, Guangzhou, China

**Keywords:** secretin, cell survival, neural development, synaptic plasticity, learning and memory

## Abstract

With emerging evidence showing a wide distribution of secretin (SCT) and its receptor (SCTR) in the central nervous system (CNS), the putative neuropeptide role of SCT has become more appreciated since the disruption of SCT/SCTR axis affects various neural functions. This mini review thus focuses on the effects of SCT on cell survival and synaptic plasticity, both of which play critical roles in constructing and maintaining neural circuits with optimal output of behavioral phenotypes. Specifically, SCT-dependent cellular and molecular mechanisms that may regulate these two aspects will be discussed. The potential complementary or synergistical mechanisms between SCT and other peptides of the SCT superfamily will also be discussed for bridging their actions in the brain. A full understanding of functional SCT/SCTR in the brain may lead to future perspectives regarding therapeutic implications of SCT in relieving neural symptoms.

## Introduction

The gastrointestinal functions of secretin (SCT) has been recognized for more than one century since it was initially noted for its role in facilitating pancreatic exocrine secretion of bicarbonate-rich fluid (Bayliss and Starling, 1902). However, the central importance of SCT has been gradually appreciated only during the last four decades. One piece of the pioneering proof in 1979 suggested that SCT could exert a strong stimulatory effect on cyclic adenosine monophosphate (cAMP) in neuroblastoma glioma hybrid cells (Propst et al., 1979). Later in the same year, another study further identified SCT-like bioactivity in extracts of porcine brain, thus for the first time implying the existence of SCT in the central nervous system (CNS) (Mutt et al., 1979). Since then a growing number of studies have expanded the gene expression map of SCT and its receptor (SCTR) in the brain. To date, SCT and SCTR have been found to be expressed from forebrain to hindbrain structures including cerebral cortex, hippocampus, central amygdala (CeA), thalamus, hypothalamus, pons, cerebellum, medulla oblongata and nucleus of the solitary tract (NST) [reviewed in Wang R. et al. (2019)]. In a species-dependent manner, SCT and SCTR have been recognized in human (Carlquist, 1985; Chow, 1995), mouse (Lan et al., 1994; Vassilatis et al., 2003), rat (Gossen et al., 1989; Ishihara et al., 1991), rabbit (Gossen et al., 1990; Svoboda et al., 1998), and many other mammalian species (Nilsson et al., 1980; Mats et al., 1981; Shinomura et al., 1987; Buscail et al., 1990; Bounjoua et al., 1991). Notably, the amino acid sequence of mature SCT and SCTR peptides is well-conserved across these species. Taken together, the wide distribution of SCT and SCTR throughout the brain and the high degree of sequence conservation among species suggest their biological significance. The key question is what roles does SCT have in the CNS under normal and pathological conditions? To further elaborate the neurological functions of SCT, researchers have been working on the development of SCT and SCTR gene knockout mouse models. Using those models in multiple biological and/or behavioral tests thus provides strong genetic support for the functional diversity of SCT in the CNS [reviewed in Zhang and Chow (2014)], which primarily includes the role of hippocampal SCT in social recognition and spatial memory, regulation of water homeostasis (Chu et al., 2007, 2009) and food intake by hypothalamic SCT (Cheng et al., 2011), and cerebellar SCT-mediated motor coordination and motor learning (Zhang et al., 2014). Moreover, SCT has been implicated in certain neurodevelopmental disorders such as autism and schizophrenia (Alamy et al., 2004; Toda et al., 2006). In this mini review, we will discuss current evidence for its specific effects on cell survival and synaptic plasticity in the CNS.

## Effects of SCT on Cell Survival and Neural Development

The well-coordinated interplay between neuronal death and survival constitutively occurs during development of the CNS. On one hand, apoptosis is required to maintain the adequate neuronal population by eliminating excess neurons, achieving a “quality-control” process to remove developmental errors. On the other hand, survival of neural progenitor cells and new-born neurons is required to maintain normal neurogenesis and further neural plasticity within the adult brain (Meier et al., 2000). One of the most extensively studied effects of SCT resides in its neuroprotective potency against apoptosis and in favor of cell survival. Physiologically, SCT deficiency results in excess apoptosis in the dentate gyrus (DG) of hippocampus (Jukkola et al., 2010) and the external granular layer (EGL) of cerebellum during early postnatal development (Wang et al., 2017). Using *in situ* Terminal deoxynucleotidyl transferase (TdT) dUTP Nick-End Labeling (TUNEL) assay, more apoptotic cells were found in these two subregions of SCT knockout mice where neural progenitor cells reside and undergo intensive proliferation. However, when the proliferation of neural progenitor cells was examined, there was no significant difference in the number of 5-ethynyl-2′-deoxyuridine (EdU)-incorporated new-born neurons between SCT-deficient and wild-type mice (Wang et al., 2017). Under pathological conditions such as ethanol exposure at early postnatal age, the number of apoptotic cells in the EGL of cerebellum as well as in the striatum was obviously increased in both SCTR knockout and wild-type mice, but the increase was much more significant with SCTR deficiency (Hwang et al., 2009). These findings thus indicate that SCT and SCTR are necessary for the survival, but not proliferation, of neuronal progenitors in both physiological and pathological CNS.

In addition, the survival of new-born neurons also requires intact SCT/SCTR signaling. In the hippocampal DG, the total number of EdU-labeled new-born cells surviving after 3 weeks was remarkably reduced in SCT-deficient mice (Jukkola et al., 2010). In the cerebellum, newly generated granular cells in the EGL progressively migrate inward to reside within the destined positions of the internal granular layer (IGL) where further maturation follows. Similar phenotypes also occur in the cerebellar IGL where s higher number of apoptotic cells was found in SCT knockout mice than that in their wild-type littermates (Wang et al., 2017). Based on current knowledge, however, it is still unclear whether the poor survival rate of granular cells is due to the lack the neurotrophic factors that are needed for survival, or due to the deficits for their inability to establish appropriate synaptic projections with target neurons as a consequence of their premature migration (Wang et al., 2017). It is worth noting that the density of cerebellar Purkinje cells also decreased under SCT deprivation. Such phenotype appears to depend on a cell-autonomous effect of SCT as the conditional knockout of SCT in Purkinje cells gave rise to a comparable reduction of Purkinje cell density (Wang et al., 2017).

During the later stage of neural development, intact dendritic arborization is equally necessary to ensure optimal structure and functionality of the CNS (Valnegri et al., 2015). We recently found prominently impaired dendritic arborization as displayed by fewer branches and shorter lengths in Purkinje cells of SCT knockout mice. The density of their dendritic spines was also dramatically decreased in SCT knockout mice, suggesting a neurotrophic role of SCT in the cerebellum (Wang et al., 2017). However, SCT or SCTR deprivation did not affect dendritic morphology in hippocampal CA1 pyramidal neurons, whilst SCTR deficiency did reduce dendritic spines in the first order apical dendritic branches of those pyramidal neurons (Nishijima et al., 2006; Yamagata et al., 2008). We thus consider the possibility that SCT may exert a preferential or specific influence over the dendritic and spine development across different brain regions. Moreover, such impairments in dendritic arborization are thought to disrupt its wiring with presynaptic boutons, thus adversely affecting synaptic transmission and plasticity, leading to behavioral deficits.

Although the data reviewed above indicate the necessary role of intact SCT/SCTR axis in the CNS for cell survival and neural development, the understanding for its molecular mechanisms is far from complete. In general, SCT binding triggers two distinct signaling pathways *via* activation of adenylyl cyclase (AC) and phospholipase C (PLC). As the downstream effector, AC initiates an intracellular accumulation of the secondary messenger cAMP and the subsequent activation of cAMP-dependent protein kinase A (PKA), while PLC catalyzes the production of two secondary messengers, inositol 1,4,5-trisphosphate (IP_3_) and diacyl glycerol (DAG) to induce Ca^2+^ release from endoplasmic reticulum and to activate protein kinase C (PKC), respectively. We thus believe that the contribution of SCT/SCTR signaling to neuronal survival and development is probably associated with those molecular pathways. Our recent studies have proposed a schematic diagram revealing the signaling pathways involved in neuroprotective effect of SCT in the cerebellum (Wang et al., 2017; Wang L. et al., 2019). Using *ex vivo* cerebellar slice culture combined with pharmaceutical manipulation, we found that SCT induced phosphorylation of cAMP response element binding protein (CREB) largely by cAMP/PKA signaling pathway (Wang et al., 2017). As the common downstream target effector of multiple survival pathways including PI3K/Akt, MAPK/ERK, and cAMP/PKA pathways, CREB serves as one transcription factor to up-regulate anti-apoptotic proteins such as Bcl-2 and Bcl-xL (Finkbeiner, 2000). Further examinations found that SCT-induced CREB activation was also dependent on extracellular signal regulated kinase 1/2 (ERK1/2) but not Akt (Protein Kinase B), and that only concurrent suppression of both PKA- and ERK-dependent pathways can effectively abolish the anti-apoptotic effect of SCT (Wang et al., 2017). A later study also showed that PKA- and ERK-dependent CREB signaling contributed to the effect of SCT on mediating Bcl-2 and Bcl-xL expression *via* a synergistical manner (Wang L. et al., 2019). Consistently, the activity of those critical signaling molecules were all strikingly reduced in the cerebellum of SCT-deficient mice (Wang et al., 2017; Wang L. et al., 2019). Here in terms of SCT-induced ERK1/2 phosphorylation, it was also partially inhibited by the presence of PKA inhibitor, suggesting the participation of both cAMP/PKA-dependent and -independent signaling pathways. These results thus add more complexity for elucidating the mechanisms underlying neuroprotection of SCT. In addition to the cerebellum, the cAMP/PKA/CREB pathway has also been found to be involved in the neural actions of SCT within the hypothalamus (Mak et al., 2019) and CeA (Pang et al., 2015). Therefore, we may expect that such molecular mechanisms also play a role for anti-apoptotic effects of SCT in many other areas of the CNS.

**FIGURE 1:**
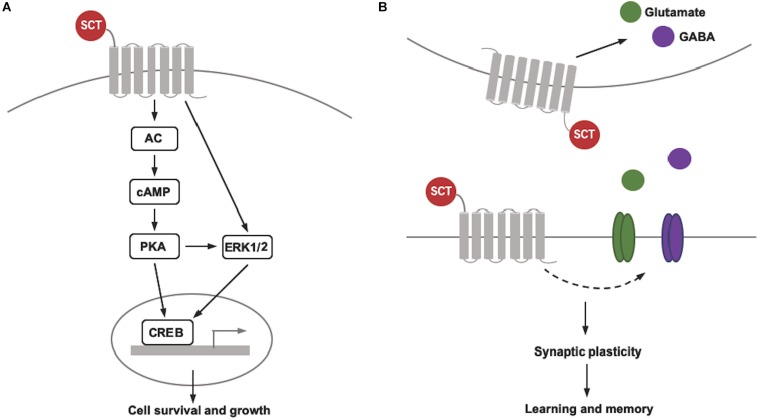
Schematic diagram showing SCT’s pleiotropic actions on cell survival and synaptic plasticity. **(A)** SCT/SCTR signaling pathway that contributes to the cell survival and growth. **(B)** Bidirectional regulation of synaptic plasticity of SCT *via* different pre- and post-synaptic mechanisms.

So far, few researchers have been working on the mechanisms underlying SCT’s neurotrophic effects. One early *in vitro* study demonstrated that SCT promoted both the number and length of neurites in cultured pheochromocytoma PC12 cells through PKA-ERK1/2 pathway (Kim et al., 2006). Notably, CREB is also known to mediate dendritic morphogenesis through transcriptional activation (Redmond et al., 2002). Therefore, it is possible that SCT stimulates dendrite growth and spine formation through similar signaling pathways as proposed above, although evidence is warranted for supporting this notion. In summary, our current findings illustrate that diverse molecular mechanisms synergistically contribute to SCT’s neuroprotective role in the cerebellum (Figure 1A), providing clues for understanding potential signaling pathways by which SCT controls neural functions. Further studies are required to investigate how these pathways interact and converge to modulate specific roles of SCT.

## Effects of SCT on Synaptic Plasticity and Memory

Long-term potentiation (LTP) and long-term depression (LTD) are two critical processes that underlie long-term synaptic plasticity. Both are long-lasting changes in synaptic strength resulting from specific patterns of synaptic activity, and are considered as putative synaptic mechanisms contributing to learning and memory (Citri and Malenka, 2008). To date, only limited research has been conducted to investigate the role of SCT/SCTR signaling in regulating synaptic plasticity, although animal experiments have clearly shown its essential role in learning and memory. SCTR-dependent LTP was first noted at the hippocampal Schaffer collateral to CA1 pyramidal neuron (SC-CA1) synapse (Nishijima et al., 2006), a neural circuit that has been well-studied as a key component for hippocampal-dependent memory encoding. Utilizing the SCTR-knockout mouse model, the authors found that a high-frequency stimulation (two trains at 100 Hz for 1 s separated by 20 s) at the SC-CA1 synapse failed to induce an apparent LTP of population excitatory postsynaptic potentials (pEPSPs). In specific, both the induction and maintenance of LTP were significantly impaired in SCTR-deficient mice (Nishijima et al., 2006). A consistent phenotype of LTP deficit was later obtained in SCT-knockout mice, which also showed a remarkable decrease in LTP induction and maintenance compared to their wild-type controls (Yamagata et al., 2008). In conjunction with the prominent expression of SCT and SCTR in the hippocampal CA1 region (Nishijima et al., 2006; Yamagata et al., 2008), these findings collectively indicate that intact SCT/SCTR signaling is needed to induce normal LTP in the CA1 area of the hippocampus. More importantly, as a consequence of LTP dysfunction at the SC-CA1 synapse, SCT-knockout and SCTR-knockout mice exhibited behavioral deficits of spatial learning in the water maze task and social recognition memory in the partition test (Nishijima et al., 2006; Jukkola et al., 2010), highlighting the functional significance of SCT/SCTR signaling-mediated synaptic plasticity.

The effect of SCT on synaptic plasticity and memory has also been implicated in the cerebellum. In rats, infusion of SCT into the cerebellar cortex facilitated the acquisition of delay eyeblink conditioning (EBC), a classical cerebellum-dependent motor learning behavior, while intracerebellar infusion of SCTR antagonist exerted the opposite effect and neither of the infusions significantly affected the extinction phase of delay EBC (Williams et al., 2012; Fuchs et al., 2014). These two separate studies from the same research group demonstrate the activation of SCTR in the cerebellum by both exogenous and endogenous SCT during the learning process of EBC. Moreover, motor learning deficits have been observed in mice lacking SCT or SCTR. In particular, when SCT gene is specifically deleted from cerebellar Purkinje neurons, significant learning deficits in the accelerating rotarod test were observed in those transgenic mice (Zhang et al., 2014). These findings from different mouse models thus add profound evidence for the functional role of cerebellar SCT in motor skill learning. To provide mechanistic explanations, further studies are still needed to directly investigate the effect of SCT on synaptic plasticity of cerebellar circuits, which can be linked to these behavioral changes. It has been growingly believed that different forms of plasticity in the cerebellar cortex operating in a distributed and synergistic manner underlie motor learning (Gao et al., 2012). For example, as supported by a recent study, EBC is dependent on both LTD at the parallel fiber-Purkinje cell (PF-PC) synapse and feed-forward inhibition of molecular layer interneuron-Purkinje cell (MLI-PC) transmission with both mechanisms compensating for each other’s disruption (Boele et al., 2018). As the presynaptic modulation, SCT may induce endogenous release of glutamate from the cerebellum and facilitate GABA release from presynaptic basket cell terminals onto postsynaptic Purkinje cells (Yung et al., 2001; Lee et al., 2005). Meanwhile, on the postsynaptic side, SCT potentiate the inhibition of Purkinje cells by reducing surface expression of Kv1.2 at basket cell-Purkinje cell synapses and in Purkinje cell dendrites (Williams et al., 2012; Fuchs et al., 2014). In addition, SCT-induced glutamate release and surface Kv1.2 reduction may also facilitate PF-PC LTD. These findings suggest that SCT has potential in mediating different forms of cerebellar cortical plasticity.

In contrast to the improvement of hippocampus- and cerebellum-related memory, SCT suppresses conditioned fear memory as demonstrated by the decreased magnitude of conditioned fear-induced startle response in rats following peripheral administration (Myers et al., 2004). Such inhibition of fear conditioning by SCT was thought to depend upon amygdala, a brain site with a critical role in the acquisition and expression of conditioned fear memory. Using an *in vitro* autoradiography technique, one previous study has reported moderate SCT binding in the CeA (Nozaki et al., 2002). SCT and SCTR mRNA expression in the CeA was also detected by quantitative real-time PCR (Yang et al., 2004). As functional evidence, both peripheral and central injection of SCT induced intensive expression of the immediate-early gene c-Fos in the CeA of rats (Goulet et al., 2003; Welch et al., 2003). More specifically, local microinjection of SCT into the CeA has been recently revealed to modulate spontaneous firing of CeA neurons (Pang et al., 2015). In particular, consistent with these animal data, intravenous administration of SCT into human clearly increased the amygdala activation in response to fear stimuli (Yurgelun-Todd et al., 2008), supporting the idea that SCT may modulate amygdala activity and synaptic plasticity during fear learning and memory. Taken together, SCT has emerged as a pleiotropic neuropeptide to regulate the bidirectional long-term synaptic plasticity and thereby regulate learning and memory functions (Figure 1B). However, it is still in need of more research to clarify the specific role of SCT in controlling different forms of plasticity and to illustrate their underlying cellular and molecular mechanisms.

## Conclusion and Future Perspectives

Some striking results on the central roles of SCT have been obtained in the past 40 years. Here we mainly reviewed the involvement of SCT in the control of cell survival and synaptic plasticity and thereby in the regulation of neural development and memory process as summarized in Table 1. The phenotypes of SCT and SCTR knockout mice are generally consistent despite of the differences of their cell-specific expression within brain areas, suggesting SCT’s pleiotropic actions on cell survival and synaptic plasticity are exerted by specifically binding to SCTR. Future studies should be performed to explore the cell-autonomous and non-autonomous mechanisms of SCT so as to gain a more comprehensive understanding on SCT’s functional profiles.

**TABLE 1:** Cellular distribution of SCT/SCTR and their effects on cell survival and synaptic plasticity in specific brain regions: implications for learning and memory.

	Hippocampus	Cerebellum	Amygdala
SCT/SCTR expression	SCT in dentate gyms (DG), hilus, molecular layer (Yamagata et al., 2008); SCTR in CA1 (Nishijima et al., 2006)	SCT in Purkinje neuron, deep cerebellar nuclei (DCN) (Yung et al., 2001; Zhang et al., 2014); SCTR in Purkinje neuron, basket cell, granular cell progenitor (GCP, during postnatal development) (Yung et al., 2001; Wang et al., 2017)	SCT and SCTR in central nucleus of the amygdala (CeA) (Nozaki et al., 2002; Yang et al., 2004)
Cell survival and neural development	Reduced survival of neural progenitor cells and new-born neurons in the DG of SCT knockout mice (Jukkola et al., 2010); Reduced number of dendritic spines in CA1 pyramidal neurons of SCTR knockout mice (Nishijima et al., 2006)	Increased apoptosis in the external granular layer (EGL) and internal granular layer (IGL) of SCT knockout mice (Wang et al., 2017); Significant ethanol-induced apoptosis in the EGL of SCTR knockout mice (Hwang et al., 2009); Reduced Purkinje cell number in SCT knockout and Purkinje cell-specific SCT knockout (Pur-Sct^–/–^) mice (Zhang et al., 2014; Wang et al., 2017) Impaired dendritic arborization and reduced spine density in the Purkinje neurons of SCT knockout mice (Wang et al., 2017)	N/A
Synaptic plasticity	Decreased LTP induction and maintenance at the Schaffer collateral-CA1 (SC-CA1) synapse in SCT and SCTR knockout mice (Nishijima et al., 2006; Yamagata et al., 2008)	Putative SCT-induced LTD at the parallel fiber-Purkinje cell (PF-PC) synapse (Lee et al., 2005; Williams et al., 2012)	N/A
Behavioral phenotypes	Impaired spatial learning ability in SCT knockout mice (Jukkola et al., 2010); Impaired spatial learning and social recognition behaviors in SCTR knockout mice (Nishijima et al., 2006)	SCTR-dependent acquisition of delay eyeblink conditioning (EBC) (Williams et al., 2012; Fuchs et al., 2014) Motor learning deficits in SCT knockout, SCTR knockout and Pur-Set^–/–^ mice (Zhang et al., 2014)	SCT-inhibited conditioned fear memory (Myers et al., 2004)

In phylogenetic analysis, SCT is categorized into a peptide superfamily, which also consists of vasoactive intestinal peptide (VIP), pituitary adenylate cyclase activating polypeptide (PACAP) and many other members with particular importance in the CNS. Interestingly, SCT and these neuropeptides have been found to share some overlapping neural functions. For example, both PACAP and VIP can act as a powerful neuroprotective factor and promote cell survival through cAMP signaling pathways with direct modulation on Bcl-2 (Gutiérrez-Cañas et al., 2003; Castorina et al., 2008). Additionally, PACAP-deficient and PAC1 receptor-deficient mice also showed reduced hippocampal LTP and impaired hippocampus-dependent recognition memory and associative learning (Otto et al., 2001; Matsuyama et al., 2003; Takuma et al., 2014). Therefore, we propose that SCT may work with different neuropeptides in a complementary or synergistical manner to fine-tune the behavioral output of neural circuits across different brain regions. Indeed, a recent study has found that receptors of SCT and glucagonlike peptide-1 (GLP-1), another member of SCT superfamily, are able to form heteromer in cells coexpressing these two receptors. The heteroreceptor complexes mediated cell responses to SCT by reducing intracellular calcium and inducing the cointernalization of both receptors, and as a result may also bring functional alterations to stimulatory actions of GLP-1 (Harikumar et al., 2017). This illustrates that SCT and GLP-1 can achieve some combinational effects *via* heterodimerization of their receptors. Meanwhile, while sharing functional similarities on stimulating insulin secretion, SCT and GLP-1 have the opposite roles in the regulation of water intake (Lee et al., 2010; McKay et al., 2014). Some consideration should be still given to the distinct actions and mechanisms of each peptide. Combination of the beneficial effects of SCT and its cousin peptides might hopefully improve multiple biological activities and thereby achieve optimal therapeutic outcomes.

Both neuronal loss and deficits in long-term synaptic plasticity are pathological features in various neurodegenerative disorders such as Alzheimer’ s disease, Parkinson’ s disease, and Huntington’s disease. With the potency of promoting cell survival and modulating synaptic plasticity, SCT or its analogs may serve as a therapeutic agent targeting those neurological diseases. Future studies can be performed to test the effects of SCT infusion or pharmacological activation of SCTR in animal models with brain pathologies and cognitive disability. The elucidation of the cellular and molecular mechanisms underlying SCT neural functions may provide insights for precise intervention and pharmaceutical development.

## Author Contributions

LW and LZ wrote and revised the manuscript.

## Conflict of Interest

The authors declare that the research was conducted in the absence of any commercial or financial relationships that could be construed as a potential conflict of interest.
